# Performance Analysis in Padel: A Systematic Review

**DOI:** 10.5114/jhk/168640

**Published:** 2023-07-06

**Authors:** Iván Martín-Miguel, Adrián Escudero-Tena, Diego Muñoz, Bernardino J. Sánchez-Alcaraz

**Affiliations:** 1 Department of Musical, Plastic and Corporal Expression, Faculty of Sport Sciences, University of Extremadura, Cáceres, Spain.; 2 Department of Physical Activity and Sport, Faculty of Sport Science, University of Murcia, Murcia, Spain.

**Keywords:** racquet sports, temporal structural, game structure

## Abstract

The aim of the study was to carry out a systematic review of the most recent research on performance analysis in padel. An electronic search was made in four sport science databases: Web of Science, Pubmed, Scopus and Google Scholar. Systematic review principles were used to identify and select studies following inclusion and exclusion criteria. From a total of 261 articles identified in the initial search, 27 articles were included in analysis, all dating from after 2018. The articles were classified according to four study variables: temporal aspects, game actions, on-court movements and match score studies, ordered in turn according to the year of publication. The results show differences in the four study variables according to the gender or the level of players, the side or the zone of play and the duration of the match. In conclusion, the results define the relevant aspects of the game with the aim of being used at a technical, tactical and physical level, as well as contributing to the development of scientific knowledge in padel, allowing future research to address less studied topics and carry out more complete and specific studies and interventions for a greater understanding of the needs of padel.

## Introduction

Padel is a racket sport that was born in Mexico in 1969 (Sánchez-Alcaraz, 2013). In recent years, it has experienced an exponential increase in the number of practitioners in Spain (Courel-Ibáñez et al., 2017a) both at a social level, as it increases the level of well-being in practitioners (Villena-Serrano et al., 2020), and at a professional level due to an increase in the number of federative licenses (Gómez et al., 2019).

Padel is played on a court measuring 20 x 10 m, surrounded by glass walls or walls and electro-welded mesh, 4 and 3 m high (International Padel Federation, 2020), with the ball being able to bounce off the side or back walls, with this interaction accounting for a quarter of the total actions of the game (Gea García et al., 2021). Similarly, it is a high-intensity intermittent sport that combines high-frequency and low-intensity gestures (Sánchez-Muñoz et al., 2020), which in turn leads to an increase in the pace of play and participation by players (Courel-Ibáñez et al., 2019) compared to other racket sports that do not have this characteristic. Cardiorespiratory factors, strength and agility are considered key to optimise padel performance. Especially agility, due to changes of direction during play (Pradas et al., 2021), is an important factor to evaluate as it determines adaptations to training (De Villarreal et al., 2023).

The increase in the number of players has also led to a greater number of scientific publications in this field (Sánchez-Alcaraz et al., 2015), mostly those related to psychology (Díaz-García et al., 2021, 2023), physiology, medicine and especially performance analysis (Sánchez-Alcaraz et al., 2018, 2023a), defined as the investigation of performance in competition and training in sports in different contexts (O'Donoghue, 2015). The main objective of performance analysis is to analyse, record and evaluate the game actions and behaviors of players in real game situations (Courel-Ibáñez et al., 2015) in order to be used later by coaches.

Performance research in padel has focused in recent years on four fundamental areas: temporal structure (García-Benítez et al., 2018; Sánchez-Alcaraz et al., 2019, 2021a; Zırhlı and Demirci, 2020), game actions (Courel-Ibáñez et al., 2017b; Escudero-Tena et al., 2022b; Sánchez-Alcaraz et al., 2020c, 2022b; Ramón-Llin et al., 2020b), player movement (Ramón-Llín et al., 2020a, 2020c) and scoreboard study (Escudero-Tena et al., 2022b; Muñoz et al., 2022; Sánchez-Alcaraz et al., 2019, 2021b). In turn, in recent times there have been different systematic reviews that focused on anthropometric and physiological variables of players, injury risk and rehabilitation strategies (Demeco et al., 2022), biomechanical, physical and physiological factors (García-Giménez et al., 2022), physiological demands, body compositions, the level of fitness, and injuries (Sánchez-Alcaraz and Courel-Ibáñez, 2022) as well as padel requirements such as predominant game actions, player movements and time structure (Sánchez-Alcaraz et al., 2018). There has been only one study that focused on game actions, time structure, player movement and scoreboard study (Sánchez-Alcaraz et al., 2018), but it did not take into account the latest research in this regard and how the game has evolved in recent years.

Therefore, the aim of this work was to carry out a systematic review of the most recent research on performance analysis in padel. These results would allow to define the most relevant aspects that define a better performance in a padel match, so that coaches and players can develop competition strategies, carry out specific training tasks and provide feedback for better decision making and greater sporting performance.

## Methods

### Study Design

The principles of systematic review (Cartwright-Hatton et al., 2004) were taken into account for this study by searching four major sport science databases (Web of Science, Pubmed, Scopus and Google Scholar) for information. To narrow the search results of the articles, the keywords ("padel" OR “paddle” OR “paddle-tennis”) AND (“rendimiento” OR “performance”) were used. The articles were screened, selecting those from the years 2018–2023. The last search was conducted on 2 January 2023.

### Inclusion and Exclusion Criteria

The following inclusion criteria were used to select the articles in this review: (a) articles published from 2018 onwards, (b) a sample of padel players, (c) original studies, (d) variables related to performance, especially focused on game actions, time structure, player movement and scoreboard study; (e) articles published in scientific journals (JCR impact index, Scimago SJR index or meeting at least 30 Latindex criteria). Excluded were (a) abstracts or communications to conferences, (b) books or book sections, (c) padel modalities such as individual padel, adapted padel or wheelchair padel. Furthermore, articles in both Spanish and English were included.

### Identification and Selection of Studies

The search process was conducted using the PRISMA (Preferred Reporting Items for Systematic Reviews and Meta-Analyses) method to identify potential studies to be included through six phases (Webster and Watson, 2002; Wright et al., 2007): (i) original database search, (ii) removal of duplicates, (iii) first phase screening on titles and abstracts, (iv) second phase screening on the full text of the article, (v) forward search (references cited in the included studies), and (vi) backward search (citations of the included studies).

## Results

**Figure 1:**
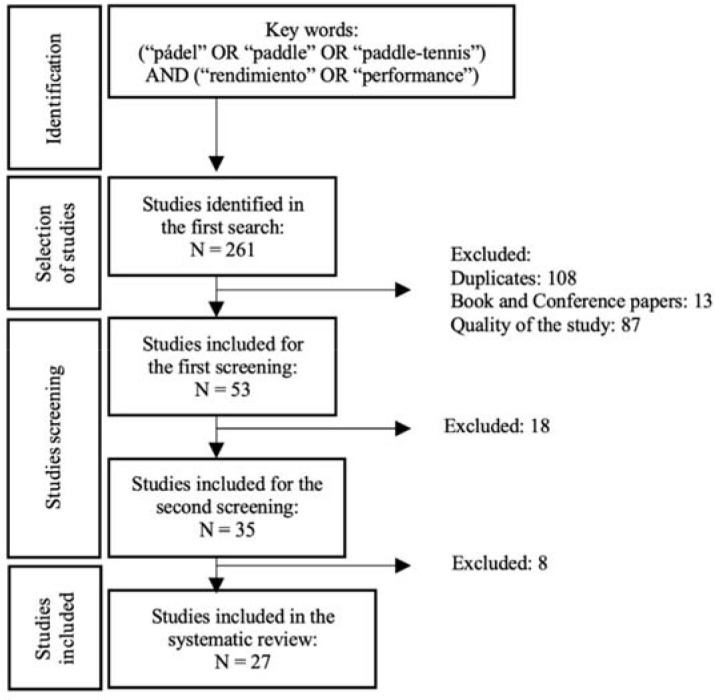
PRISMA flow diagram.

A PRISMA flow diagram with the results obtained from the information search is shown in Figure 1. The initial search provided 261 potential articles for inclusion in the review. After the first screening by analysing each title and abstract in detail, 108 were excluded because they were duplicates, 13 because they belonged to books or conferences and 87 because their quality was insufficient for inclusion. Of the 53 articles selected in the first screening, 18 were excluded after a detailed analysis of the sample used, method employed and variables considered. Finally, a total of 27 studies were included for analysis.

Table 1 shows the articles classified according to the four areas of performance analysis. The actions of play (n = 23) were the most studied topic, while the study of the match score (n = 5), temporal aspects of padel (n = 6) and players' movements (n = 2) were the areas with the least research. Regarding the year of publication, 77.7% of the papers were published from 2020 onwards. Finally, 62.9% of articles were published in international journals with the JCR impact index and 22.2% with the Scimago SJR index.

**Table 1:** Classification of articles by the thematic area of study.

Nº	Study	Temporal aspects	Game actions	Movements on the court	Match score
1	Lupo et al. (2018)	**X**	**X**		
2	García-Benítez et al. (2018)	**X**	**X**		
3	Sánchez-Alcaraz et al. (2019)	**X**			**X**
4	Ramón-Llín et al. (2019)		**X**		
5	Mellado-Arbelo et al. (2019)		**X**		
6	Courel-Ibáñez et al. (2019)		**X**		
7	Sánchez-Alcaraz et al. (2020d)		**X**		
8	Sánchez-Alcaraz et al. (2020a)		**X**		
9	Sánchez-Alcaraz et al. (2020b)		**X**		
10	Sánchez-Alcaraz et al. (2020c)	**X**	**X**		**X**
11	Ramón-Llin et al. (2020a)			**X**	
12	Ramón-Llin et al. (2020b)		**X**		
13	Ramón-Llín et al. (2020c)			**X**	
14	Escudero-Tena et al. (2020)		**X**		
15	Sánchez-Alcaraz et al. (2021a)	**X**	**X**		
16	Sánchez-Alcaraz et al. (2021b)				**X**
17	Ramón-Llín et al. (2021a)		**X**		
18	Ramón-Llin et al. (2021b)	**X**	**X**		
19	Conde-Ripoll et al. (2021)		**X**		
20	Sánchez-Alcaraz et al. (2022a)		**X**		
21	Sánchez-Alcaraz et al. (2022b)		**X**		
22	Sánchez-Alcaraz et al. (2022c)		**X**		
23	Ramón-Llín et al. (2022)		**X**		
24	Muñoz et al. (2022)		**X**		**X**
25	Escudero-Tena et al. (2022a)		**X**		
26	Escudero-Tena et al. (2022b)		**X**		**X**
27	Sánchez-Alcaraz et al. (2023b)		**X**		

**Table 1a:** Summary of performance analysis studies in padel.

Nº	Study	Sample	Variables	Main result
1	Lupo et al. (2018)	22 matches (10 female and 12 male) WPT 23 pairs analyzed (14 men and 9 women)	- Winning points - Duration of the point - Duration of the rest - Number of strokes - Type of stroke, indirect or direct - Shooting zone	Duration of the point in men's padel lower (12–13 s) than in women's padel (17 s). More strokes per point in women's padel (12) than in men's padel (9.3–9.9). Mid-court strokes predominant in men (43%) and women (54%). Smash most winning shot in boys (36.1–35.2%) and girls (37.3–30.5%). Volley second winning stroke, forehand in girls (16%), backhand in boys (16%). Forehand groundstrokes both with wall (10–11%) and without wall (11%) are predominant. The losing pair makes more groundstrokes, the winning pair makes more net shots.
2	García-Benítez et al. (2018)	1670 points from 32 national youth category players (16 male and 16 female U-16 and U-18) (15 ± 1.08 years) 12 semi-final and final matches	- Duration of the point - Rest between points - Strokes per point - Lob per point - Effective playing time - Work-rest ratio	U-16 male players have less total playing time (29.0%) compared to U-18 (34.7%). There is an average of 995 and 1185 strokes per game. Point duration is 8.9 and 12.0 s. The rest time is 14.3 and 15.5 s. There are 6.1 and 8.0 strokes per point. U-16 female players have 32.4% effective total playing time and 34.8% in U.18. In total, there are 986 points per game. Point duration is 11.3 and 11.7 s, break time 15.6, 14.1 s and there are between 6.9 and 7.2 strokes per point between U-16 and U-18, respectively.
3	Sánchez-Alcaraz et al. (2019)	701 points from 13 sets in 6 matches in the men's category of the Extremadura Padel Federation	- Playing time: short, medium and long - Rest time: short, medium and long - Difference in the score - Key moments	Playing time is 45.92% of total match time. Playing time is not influenced by the score difference or the time of the match. The difference in the score does not influence the rest time. In non-key moments the rest time is short (<10 s). At key moments the rest time is medium (10–20 s).
4	Ramón-Llín et al. (2019)	18 matches from 26 national players (33.5 ± 6.8 years) and 30 regional players (31.1 ± 6.9 years) in two tournaments	- Serve-return shot type - Ball location - Player’s position - Movement parameters	In the Australian position, returners direct 3 out of 4 returns to the server when the serve is to the glass, mainly looking for the gap in the court with a down the line return. When the serve is to the centre, there are 10% more returns intercepted by the server's partner, due to covering the centre of the court. In the traditional position, when the serve is to the “T”, 50% of the returns are hit by the server and 50% by the server's partner, while when the serve is to the glass, 10% more returns are hit by the server's partner than those hit by the server.
5	Mellado-Arbelo et al. (2019)	8581 points from 20 WPT professional players (34.5 ± 5.7 years)	- Player who hits - Location on court - Type of stroke - Trajectory of the stroke - Effectiveness - Result - Serving - Set	Backhand volley is the most used stroke (16.6%), followed by forehand volley (13.0%). 57.5% of the strokes are cross court. 49.1% of shots are from the baseline, 26.4% at the net, 19.3% in the transition zone and 0.5% outside. Smash is the stroke with which most winners are achieved (45.1%). Regarding unforced errors, they occur more with the double wall with spin (50%) and without spin (14.3%), followed by the off the wall smash (8.5%). Forced errors include the backhand back wall (30.8%) and the double wall opening with the backhand spin (26.7%). Unforced errors represent the majority of the end of points (44.9%), followed by winning points (38.4%) and forced errors (16.7%).

A summary of the sample, variables and main results of each article analyses in this systematic review is presented in Table 2.

**Table 2b:** Summary of performance analysis studies in padel.

Nº	Study	Sample	Variables	Main result
6	Courel-Ibáñez et al. (2019)	1963 hits of 4 WPT male professional players	- Type of stroke: indirect or direct - Stroke height: lob or straight - Distance from the net - Side of the court - Effectiveness - Winning stroke, error and continuity	Forehand strokes stand out over backhand strokes. Lobs account for less than 2 out of 10 groundstrokes. Baseline predominates (46.6%), followed by middle (27.7%) and net (25.6%). Lateral side is used similarly between left and right (30.5% and 32.1%), lower proportion with respect to the center (37.4%). The use of the lateral corners in the back court is 33% of the game. The use of the center in the attacking zone is 25%. The game in the middle is characterized by the use of the volley, *bandeja* and smash. The position at the net is characterized by the use of the volley. The use of the lob is 16% throughout the match.
7	Sánchez-Alcaraz et al. (2020a)	2054 strokes from 4 men's finals WPT 2017	- Forehand volley - Backhand volley - Smash - *Bandeja*	The winning pair performs a greater number of attacking actions than the losing pair (327 vs. 186) and attacking actions per point. The volley is the most used, followed by the *bandeja* and the smash. The smash is used 5% more by winners. Losers fail to achieve an attacking action in 47% of the points.
8	Sánchez-Alcaraz et al. (2020b)	668 serves and 600 serve-returns of 14 matches (7 male and 7 female) in WPT category 32 players, 16 men (age: 31.18 ± 7.27 years; body height: 181.3 ± 4.1 cm)	- Serve: the number, the side of the court, the direction of the serve and effectiveness - Serve-return: the side of the court, the direction of the serve-return, height, the type of stroke and effectiveness - Point: winners the servers or winners the receivers - Number of strokes in the point	Male players win more first serves and points on the serve. The percentage of points won is higher the fewer the number of strokes in the point. The advantage on the serve is lost after the twelfth hit in men, and seventh hit in women. More than 60% of the serves are to the side wall. From the left side, 12% more serves are to the side wall than the right side. From the right side there are 14% more serves to the “T”. 60% of the serve-return are straight shots. 90% of the returns are effective. Players on the left side serve 15% down the line more than players on the right side. Left side players execute more than 75% of their serve-return with a backhand shot. Girls use more backhand or cross court directions and the lob.
9	Sánchez-Alcaraz et al. (2020c)	24 matches (12 with advantage and 12 with golden point), 523 games (241 advantage and 282 golden point), 12 quarterfinals, 8 semi-finals and 4 WPT finals	- Tournament and round - Gender - Set, number, duration and result - Number of break balls and break made	With golden points, the set duration is shorter, the number of games increases, more breaks are made, there is a higher percentage of matched sets and 3-set matches. There is no gender difference between the match time and score. There are more break balls and the number of breaks in girls. From the semi-finals the number of games increases and the duration of the set is longer.
10	Sánchez-Alcaraz et al. (2020d)	1015 auctions of 8 WPT finals (4 men's and 4 women's), 20 players (10 men and 10 women)	- Type of smash - Effectiveness: winning stroke, error and continuity - Hitting zone: side of the court and distance from the net - Direction of the stroke: down the line or cross court	*Bandeja* is the most used shot, more in women (68.8%) than in men (55.7%). Men use the flat smash (26.6%) and the topspin (9.8%) more than women (17% and 4.9%, respectively). The off the wall smash is made more by women (9.3%) than men (7.9%). The predominant direction is down the line (52.9% in women and 59.4% in men). The middle area is the area where more strokes are made, exceeding 50% in both men and women. The off the wall smash is the shot with which most errors are made. The flat smash and the topspin offer more winners. The off the wall smash and the *bandeja* offer more continuity.

## Discussion

The aim of this paper was to carry out a systematic review of the most recent research on performance analysis in padel. After the search methodology and selection of articles, a total of 27 studies were finally included that analysed one of the four areas of performance in padel: time structure, game actions, player movement and the match score.

### Time Structure in Padel

One of the aspects most frequently addressed in research carried out in padel are the time actions of the game. Regarding the total duration of the match, studies have indicated that the duration is approximately 90 min in World Padel Tour (WPT) matches, around 40 min each set (Sánchez-Alcaraz et al., 2021a), with differences depending on gender, being higher in women (García-Benítez et al., 2018). Similarly, in both genders, set duration is shorter in matches with golden point (Sánchez-Alcaraz et al., 2020c), compared to sets played with advantage games. In relation to the level of play, WPT professional players have 30% of real play time (Sánchez-Alcaraz et al., 2021a), being lower than in regional level players (45.92%) (Sánchez-Alcaraz et al., 2019). Finally, as a function of age, the actual playing time in U-18 and U-16 players is 29.32% and 34.7%, respectively (García-Benítez et al., 2018).

**Table 3c:** Summary of performance analysis studies in padel.

Nº	Study	Sample	Variables	Main result
11	Ramón-Llín et al. (2020a)	4406 points from 27 national category padel matches	- Average distance - Time structure: active time, passive time, total playing time - Level of the player according to the ranking: low, medium and high	Players cover an average of 3000 m per game of which 50.8% is when the ball is in play. They cover an average of 11 m per point and 80 m per game. Medium level players cover more distance, by 400 m, than high-level players and 900 m more than low-level players in the active phase. Mid-level players play the most points, almost 31 more than low-level players and 22 more than high-level players. There is a positive correlation between the number of points and the distance travelled.
12	Ramón-Llin et al. (2020b)	8441 strokes and 1055 points from 24 male players in 9 matches (3 finals and 6 semi-finals) of the national category	- Type of the stroke: indirect or direct - Area of the court - Direction of the stroke - Effectiveness: winning stroke or error - Side of play	The winning pair makes more winners (5.6%) than losers (3.7%). More cross court shots are made (65.3%) than down the line (34.7%). At the net more cross court shots are made (67.5%) than down the line (61.7%). The winning pair makes more hits in the offensive zone (41.2%) than losers (35.3%). Players on the left side hit 1.9% more winners and a lower percentage of errors than those on the right side. There is a higher percentage of continuity versus winners or errors.
13	Ramón-Llin et al. (2020c)	2202 points from 18 regional category men's padel matches	- Level of play: sdvanced, initiation - Distance covered by players - Player’s role	Advanced level players cover more distance per point (11.77 m) than beginners (8.30 m). Beginner level players cover distances between 0–8 m in 60% of the points, 20% more than advanced level players. Advanced level players cover 12% more distances over 16 m. The player who serves is the one who covers more distance per point (13.61 m) followed by the returning player (12.35 m).
14	Escudero-Tena et al. (2020)	1324 baseline actions from 14 WPT women's tournament finals in 2018; Identification of 324 moves	- Effectiveness of the stroke - Reaching offensive positions - Type of hitting - Consequences of reaching offensive positions	The lob is the most used (85.4%). In most cases, the defensive pair gets offensive positions (70.4%). The defensive pair performs a lob between the second and the sixth stroke. It causes continuity in the game leading to more position changes.
15	Sánchez-Alcaraz et al. (2021a)	1042 points from 8 matches (final and semi-final) of 4 WPT tournaments; 24 players: 12 men and 12 women	- Duration of the game - Real game time - Set duration - Duration of the point - Number of strokes per point - Effectiveness: winning stroke and error	The total duration of the match is approximately 90 min. The real playing time is about 30% of the total time. Set duration is approximately 40 min. The average point duration is 12.5–13.5 s. 30% of the points have duration of 5–10 s. Girls perform 7% more points longer than 20 s. Boys make 3% more points with duration of less than 5 s. There is an average of 10 strokes per point with no difference between genders. Boys made 10% more winners and 10% fewer unforced errors.
16	Sánchez-Alcaraz et al. (2021b)	647 matches (332 men's and 315 women's); 1458 sets and 13592 WPT games in 2019	- Gender - Type of a tournament - Tournament round - Number of sets and games - Percentage of sets with tie-break - Match score	70% of the matches are resolved in the second set, with no difference between men and women. In the men's category, there are more equal sets and more tie-breaks than in the women's category, as well as more games per set and match. In the women's category, there are more matched sets in masters than in open. In the men's category, the percentage of matched sets is higher in each round, increasing in the semi-final and final. There is no difference between open and master in the set percentage, being in master where it is more equal from the semi-finals onwards.

**Table 4d:** Summary of performance analysis studies in padel.

Nº	Study	Sample	Variables	Main result
17	Ramón-Llin et al. (2021a)	1071 points from 9 games of final table 1^st^ national category of padel; male players of 33.3± 6.9 years	- Situation of the serving pair: traditional or Australian position - Serving direction: serve to the side wall or serve to the “T” - Result of the point: won by the serving or the returning pair	From the left side of the court, players hit 75% to the side wall in the Australian position and 85% in the traditional position. From the right side, they serve 23.9% more to the side wall in the traditional position. From the right side, the serving pair wins between 55 and 59% of the points. From the left side, players win 8% more when serving to the “T” using the traditional serve. Players using the traditional serve win a higher percentage of points on the serve. The number of points won on the serve decreases as the match progresses. Australian strategy loses 6% more points on the serve in the final set of the match compared to the second set.
18	Ramón-Llín et al. (2021b)	2148 points from 18 matches of two male padel indoor tournaments	- Service tactic formation - Game level - Point outcome - Movement variables	There are differences in service tactic formation according to the player’s level. Beginners use the traditional position in more than 80% of the points. High level players use both tactical positions interchangeably, but preferably the Australian position. The serving pair wins more points than returners in both levels. More than 70% of the rallies are resolved in the first 9 s. More points are earned with the traditional tactic position.
19	Conde-Ripoll et al. (2021)	4665 strokes from 12 matches (7 men's and 5 women's) WPT in the quarterfinal, semi-final and final rounds	- Number of walls - Hitting with or without rotation - Type of stroke: indirect, direct or resource - Side of the stroke - Stroke height - Effectiveness: winning stroke, error and continuity	80% of backstrokes are with one wall. The backwall shot is the predominant stroke (+80%), followed by off the wall (+10%). The predominant side of the body is the forehand with 13% more than the backhand. 17% more winners are obtained with the forehand and 17% more errors with the backhand. Straight strokes (51%) are similar to lobs (49%). 90% of groundstrokes provide continuity. There are 5% more errors than winners. 30% more winners are made when the ball comes from the back wall. When the ball is side wall or double wall the error percentage increases.
20	Sánchez-Alcaraz et al. (2022a)	633 actions of 8 matches (4 finals and 4 semi-finals) WPT; 12 men and 12 women	- Type of the stroke: volley, flat smash, *bandeja* - Effectiveness of the stroke: point won and lost - Distance from the net	The flat smash is performed more by men (37.7%) than women (29.2%). *Bandejas* are made more by women (23.3%) than men (11.7%). 60% of attacking shots to finish are between 2–6 m and 15–18% between 6–8 m. Flat smashes are performed by women (+70%) between 0–4 m and men (65%) between 4–8 m. *Bandejas* are made between 65 and 70% and between 6 and 8 m. 70% of volleys are made between 2 and 4 m. 35% of volleys are winners. Zone 0–6m winning percentage is higher than the error. Zone 8–10 m error percentage is higher than the winner.
21	Sánchez-Alcaraz et al. (2022b)	489 strokes of 14 matches (7 male and 7 female) in the professional category	- Player in net - Type of the stroke: indirect or direct - Effectiveness: winning strokes, errors and not definitive - Hitting side - Distance from the net - Hitting trajectory	There is a 26% increase in the number of balls to the fence by serving players. 45% of the hits to the fence come from the center of the court, 10% more are made by the player on the right side. There is 60% continuity. Half of the shots to the fence occur in the first 10 strokes. Men make 9% more volleys and women 9% more overhead shots. On definite points, there is an 8% increase in winning shots to the fence compared to non-definite. Cross court strokes have an 8% higher chance of winning than down-the-line. Down-the-line shots have a 23% more chance of error than cross strokes.

**Table 5e:** Summary of performance analysis studies in padel

Nº	Study	Sample	Variables	Main result
22	Sánchez-Alcaraz et al. (2022c)	1927 points from 14 matches (7 men and 7 women) WPT, the last shot was counted, 654 winners and 1273 errors, made by 32 players (16 women and 16 men)	- Effectiveness: winning shot and error - Area of the court - Result of the match - Winning and losing pair - Importance of the point - Type of stroke: indirect or direct	The smash is the stroke that produces the most winning points (43%) along with volleys (28.4%), While the lob is the one that produces the least (0.03%). Groundstrokes are the strokes with most errors (30.2%), followed by volleys (30.2%) and lobs (14.8%). Lobs and groundstrokes have more errors than winners and smashes more winners than errors. 56% of the winning shots are made at the net. 42% of the errors are made on the baseline. Winning pair achieves 20% more winners than errors. Losing pair commits 12% more errors than winning shots.
23	Ramón-Llín et al. (2022)	2110 game actions corresponding to the penultimate and last stroke, 1055 points from 9 games of the first Spanish national category of male players	- Court area - Stroke: number and direction - Type of stroke: indirect or direct - Effectiveness: winning stroke, error and continuity - Side of play - Result of the match	64.1% of the penultimate stroke is on the baseline and the last stroke, 60% is on the net. There are 20% more cross court strokes than down-the-line shot in the last two strokes of the point. There is 10 and15% more participation of the player on the left in the penultimate and the last stroke of the point, respectively. At the net, 82.7% of the winning points are hit against 46.5% of the total errors. The winning pair makes 48.1% of errors in the baseline and the losing pair makes 58.4%.
24	Muñoz et al. (2022)	419 sets and 838 games from 100 men's and 74 women's matches from 15 WPT tournaments	- Game round - Gender - Number of sets and game - Percentage of games decided by the golden point - Result of the match - Number of gold points won	In men's padel, the number of games in the second set is higher. The number of gold points in the second set is higher than in the third set regardless of the round. The winning pair earns more gold points. Girls win more gold points than boys. In the female category there is more equality in the score and a higher percentage of golden points. There is no difference in the gold point per round and match, but there is a difference in the percentage of games decided by the gold point. As the rounds progress, the men's padel is more equal with respect to the set score.
25	Escudero-Tena et al. (2022a)	5513 points (2645 men's and 2868 women's) corresponding to 91 sets from 38 WPT quarterfinals, semi-finals and finals	- Category: male and female - Type of finishinner and error - Type of the stroke - Number of strokes	Men make 51.6% winners, with the smash (30.6%), forehand volley (14.2%) and backhand volley (15.6%) winning the most. Women get 48.4% of winners with the forehand volley (21.9%), smash (20.7%) and *bandeja* (15.7%). Male errors total 42.6%, the backhand volley (17.6%) and forehand (17.0%) are predominant. Women make 57.4% of errors with the *bandeja* (16.5%) and forehand volley (15.3%) as predominant. More points are won between the 6^th^ and the 10^th^ stroke, the longer the point the probability of winning decreases. Between the stroke 1 and 5, more errors are made, yet their number decreases as the number of strokes increases.
26	Escudero-Tena et al. (2022b)	2759 points (1432 male and 1327 female) from 395 matches in six World Padel Tour tournaments	- Gender - Importance of the point - Type of the stroke: indirect or direct - Effectiveness: winning stroke and error	The smash is the most frequent last shot in men's padel (36.6%) and women's padel (31.8%). In key moments, boys make more *bandejas* and smashes than groundstrokes. Boys make more errors and more winning points. More errors are made on the golden point in groundstrokes. More errors are made in non-key and key moments, but less in the golden point. In both genders, the most common errors are wall strokes. More errors are made than winning shots in both genders.

**Table 6f:** Summary of performance analysis studies in padel

Nº	Study	Sample	Variables	Main result
27	Sánchez-Alcaraz et al. (2023b)	3334 strokes of the first four games of 14 matches (7 men and 7 women) WPT analyzing 32 players (16 men and 16 women)	- Gender - Forehand or backhand player - Result of the match - Zone of the court - Side of the court - Type of stroke: indirect or direct	In the net zone, around 20% of the strokes are executed in the central zone. In the middle and baseline, 20% more strokes are executed in the side areas. The backhand player makes more strokes in his zone of the court than the forehand player at the net. Winners make 5% more hits at the net.

On the other hand, the duration of points is another important aspect to take into account when planning efforts in training and matches. The duration of point in professional players is between 12.5 and 13.5 s (Sánchez-Alcaraz et al., 2021a). There are also differences according to age, being lower in U-18 (8.9 s) and U-16 (12 s) players (García-Benítez et al., 2018) and based on gender, being lower in male padel (12–13 s) compared to female padel (17 s) (Lupo et al., 2018). However, considering the distribution of the duration of the points, almost 50% last less than 10 s (Sánchez-Alcaraz et al., 2021a).

Rest interval duration, together with the duration of the point, is an important aspect to be taken into account by players in order to control their efforts. In padel, rest intervals are determined in the rules, being 20 s between points and 90 s for side changes at the end of odd games (International Padel Federation, 2008). Rest intervals are not influenced by the difference in the score (Sánchez-Alcaraz et al., 2019), nor according to age and gender, being on average between 14–15.5 s (García-Benítez et al., 2018). On the other hand, there are differences depending on the importance of the point as in the non-key moments of the match, rest intervals are less than 10 s, and in the key moments of the match, they range between 10 and 20 s (Sánchez-Alcaraz et al., 2019). As the most important practical implication, these data related to the time structure in padel can help coaches and physical trainers to design exercises and workloads adapted to the duration of points and rest intervals in padel.

### Game Actions in Padel

#### Number of Strokes

In terms of padel game actions, studies have indicated that an average of 985 and 1185 strokes are produced per match in the junior category (García-Benítez et al., 2018). In WPT matches, an average of 10 strokes per point are produced (Sánchez-Alcaraz et al., 2021a), being higher in women's (12 strokes) than in men's padel (9.3–9.9 strokes) (Lupo et al., 2018). Regarding the distribution of the number of strokes per point, in approximately 60% of the points, 10 or fewer strokes are made between the four players. However, these data vary depending on the set number, since in the third set, there is a significant increase in the number of strokes per point, increasing the percentage of points with more than 15 strokes in relation to the first and second sets (Mellado-Arbelo et al., 2019).

#### Groundstrokes

With respect to the distribution of the strokes made at baseline, 80% of the strokes are backwall strokes (Conde-Ripoll et al., 2021). In addition, forehand strokes predominate over backhand ones (Courel-Ibáñez et al., 2019; Lupo et al., 2018). The use of the lob accounts for 50% of the total shots from the back court in men's padel (Conde-Ripoll et al., 2021; Courel-Ibáñez et al., 2019), while, in women's padel, the lob is used in 85.4% of the back court shots, making it the most used to achieve offensive positions (Escudero-Tena et al., 2020) and probably being the cause of the increase in playing time and duration of the point in the women's category. In addition, game actions performed at the back of the court influence the final result of the match. It has been observed that the losing pair makes a higher percentage of strokes during a match (Lupo et al., 2018) and 10% more errors in this area of the court than the winning pair (Ramón-Llín et al., 2022).

#### Net Strokes

The shots played in this zone are mainly offensive shots. It has been observed that points scored at the net account for about 80% of the total, and winners score 34% more points than losers in this offensive zone (Ramón-Llín et al., 2022). On the other hand, it has been observed that the losing pair in a match fails to achieve an attacking action in 47% of the points (Sánchez-Alcaraz et al., 2020a). Therefore, it seems that taking up and maintaining areas close to the net guarantee success in a padel match. Regarding the predominant strokes, according to Sánchez-Alcaraz et al. (2020a), the volley is the most used shot (approximately 25% of padel strokes are volleys), while smashes are the most used stroke to win a point, both in men's and women's padel (Escudero-Tena et al., 2022b; Lupo et al., 2018). With respect to the smash, the *bandeja* (offensive stroke, without a bounce, which is made over the head and on the dominant side of the player. In this shot, before hitting the ball, the player opens the face of the racket pointing upwards and hits with a slice effect. The impact point on the ball is lower than in the other smashes (Sánchez-Alcaraz et al., 2020d)) is the most used in both women (68.8%) and men (55.7%), with the flat and topspin smash being the most used in men and the off the wall smash in women (Sánchez-Alcaraz et al., 2020d, 2022a).

#### Serve

The serve represents approximately 10% of the total number of strokes in a padel match (Sánchez-Alcaraz et al., 2020b). It also provides the possibility of taking the initiative in the point, as it allows the player to reach the offensive position of the net before their opponents. With respect to the advantage of the servers in the point, a recent study established that, as more strokes are made in the point, the percentage of points won by the serving partner decreases, establishing that, in men, from the 12^th^ stroke of the point, the advantage of being a server disappears, being in women from points with more than 7 strokes onwards (Sánchez-Alcaraz et al., 2020b). In addition, gender comparison shows that men win more points in service situations than women (Sánchez-Alcaraz et al., 2020b). However, this percentage of points won by the partner on serve decreases as the match progresses, being significantly lower in the third set (Ramón-Llin et al., 2021a) probably due to the effects of fatigue on the server, who travels the longest distance per point in professional padel (Ramón-Llin, et al., 2021a).

On the other hand, according to Ramón-Llin et al. (2021), from the right side, the serving pair wins between 55 and 59% of the points and, from the left side, they win 8% more when they serve to the T. In addition, the effectiveness of the serve seems to be influenced by the tactical position of players (Ramón-Llín et al., 2021b). The results of the studies show that players win a higher percentage of points on the serve when using the traditional position versus the Australian position, especially in the third set (Ramón-Llín et al., 2021b). This greater effectiveness of the traditional position versus the Australian position in the serve may be influenced by the fact that the use of the Australian position forces the server to travel further and faster towards the net than when using a traditional position and that, at the time of the return, he/she is at a greater distance from the net than when using a traditional position.

Regarding the direction of the serve, it also seems to be influenced by the strategy of players when serving and by the side of the court where the serve is made. In general, serving to the side wall predominates, especially when serving from the left side of the court (Sánchez-Alcaraz et al., 2020b). In addition, from the left side of the court, 10% more serves to the side wall occur in the traditional position (85%) than in the Australian position (75%) (Ramón-Llin et al., 2021a). In contrast, from the right side of the court, the directions are slightly more balanced, with approximately 60% of serves going to the side wall and 40% to the “T” (Sánchez-Alcaraz et al., 2020b). However, these data also vary depending on the tactical position of players, with 23.9% more serves to the side wall in the traditional position (Ramón-Llin et al., 2021a).

#### Return of the Serve

With respect to the return of the serve, it is a stroke that is highly effective, as in a professional padel match, players miss only 5% of the returns. Considering the characteristics of the return, in general, backhand returns predominate over forehand ones (63% vs. 37%), down the line returns over cross-court returns (70% vs. 30%) and straight returns over lobs (60% vs. 40%) (Sánchez-Alcaraz et al., 2020b). There are differences in the type of returns depending on the gender of players. In women's padel, there are 12% more backhand returns, 10% more cross-court returns and 13% more lob returns than in men's padel (Sánchez-Alcaraz et al., 2020b).

Regarding the direction of the return, players direct the return to the server or to the server's partner depending on two main factors: the direction of the serve and the position adopted by players on the serve. In the Australian position, returners direct 3 out of 4 returns to the server when the serve is to the glass, mainly looking for the gap in the court with a down the line return. However, when the serve is to the middle, there are 10% more returns intercepted by the server's partner, due to the coverage of the middle of the court. In the traditional position, when the serve is to the “T”, 50% of the returns are hit by the server and 50% by the server's partner, while when the serve is to the glass, 10% more returns are hit by the server's partner than those hit by the server (Ramón-Llín et al., 2019).

On the other hand, knowing what type of the return is more likely to be hit by opponents depending on whether they serve to the “T” or to the side wall is essential for anticipation and preparation processes. Sánchez-Alcaraz et al. (2020b) have observed how the direction (down-the-line or cross-court) and the height of the return (lob or straight) vary depending on the direction of the serve (to the “T” or to the glass). Regarding the direction of the serve-return, 66% of the serve-returns are played down the line when the serve is directed to the side wall. However, when the serve is directed to the “T”, approximately the same number of returns are down the line as cross-court. Regarding the height of the return, when the serve hits to the side wall, players perform approximately the same number of straight returns as lobs. However, when the serve goes to the “T”, almost 7 out of 10 returns are lobs, as returners are probably in a more comfortable situation when hitting and look to lob more effectively than when they are forced to return a serve to the side wall.

#### Court Areas

Several studies have divided each side of the padel court into three zones (net, middle and baseline), according to the distance to the net. The baseline and the middle court zone is where a higher percentage of strokes occur in a padel match (Courel-Ibáñez et al., 2019; Lupo et al., 2018; Mellado-Arbelo et al., 2019; Sánchez-Alcaraz et al., 2020d). However, it is in the net zone where the highest percentage of winning shots occurs, which decreases as players move away from the net (Courel-Ibáñez et al., 2019). In addition, it seems that at the back court players tend to hit closer to the corners, leaving more space in the center, while in the net zone more shots are hit in the central area, leaving more space the right and the left side (Courel-Ibáñez et al., 2019).

On the other hand, with respect to the percentage of play of the right and left side players, a similar involvement has been observed in a padel match. However, there is between 10% and 15% more involvement of the left side player in the penultimate and last shot of the point (Ramón-Llín et al., 2022; Sánchez-Alcaraz et al., 2023b), thus it seems that this player assumes more responsibility in the definition of the points. These data can serve as a reference for coaches and players for better decision making and better positioning on the court.

#### Effectiveness of Strokes

Despite the fact that padel is a sport in which as few errors as possible must be played, unforced errors represent the majority of the final points (44.9%), followed by winning points (38.4%) and forced errors (16.7%) (Escudero-Tena et al., 2022b; Mellado-Arbelo et al., 2019). In addition, girls make a higher percentage of errors (57.4%) than winning strokes (42.6%) (Escudero-Tena et al., 2022a). The effectiveness of the last shot seems to have a significant influence on the outcome of the match. The winning pair hits 20% more winners than errors and, on the contrary, the losing pair commits 12% more errors than winners (Ramón-Llin et al., 2020b; Sánchez-Alcaraz et al., 2022c).

In both genders, the most common errors occur from the baseline, with the wall strokes and groundstrokes (Escudero-Tena et al., 2022b; Sánchez-Alcaraz et al., 2022c). The forehand double wall and the off the wall smash are the ones where most unforced errors are made, while the forehand back wall and the backhand double wall are the ones where most forced errors are made (Mellado-Arbelo et al., 2019). When players are in the net area, in men's padel, the backhand volley (17.6%) is the stroke with the most errors, followed by the forehand volley (17.0%). On the other hand, in women's padel, the *bandeja* (16.5%) and the forehand volley (15.3%) are the strokes with which most errors are committed (Escudero-Tena et al., 2022a).

With respect to the winning shots, most of them are hit from the net area, and the smash is the technical gesture with which professional players win the highest percentage of points, both in men’s and women’s padel (Escudero-Tena et al., 2022a; Courel-Ibáñez et al., 2019; Sánchez-Alcaraz et al., 2022c). Regarding the type of the smash, men win a higher percentage of points with the flat and topspin smash, while women use more of the *bandeja* (Sánchez-Alcaraz et al., 2020d, 2022c). Finally, cross-court shot trajectories seem to be more effective in winning the point or producing the opponent's error than down the line trajectories (Ramón-Llin et al., 2022).

#### Direction of Strokes

In relation to the direction of strokes in padel, in general terms, 57.5% of the strokes are cross-court (Mellado-Arbelo et al., 2019). In addition, 20% more cross-court strokes occur in the last two strokes of the point (Ramón-Llín et al., 2022). Regarding the side of play, backhand players play more cross-court shots than forehand players (65.8% vs. 61.9%). Considering the area of the court, in the net area more cross-court shots are hit (67.5%) than in the baseline (61.7%) (Ramón-Llin et al., 2020b). However, with the smash, the predominant direction is parallel versus cross-court, both in men's (59.4% vs. 40.6%) and women's padel (52.9% vs. 41.1%) (Sánchez-Alcaraz et al., 2020d).

### Movement of Players

In relation to players' movements around the court, advanced padel players cover an average of 3000 m per match, of which 50.8% are done when the ball is in play (Ramón-Llín et al., 2020a). Overall, in approximately 40% of padel points, players cover distances of less than 8 m. However, it has been observed that these values of the distance covered vary depending on variables such as the score, the duration of the match, and the performance level of players. When players are at a higher level or the score is more even, the distance covered by players increases. Advanced level players cover approximately 4 m more per point than amateur players (11.77 m vs. 8.30 m) (Ramón-Llín et al., 2020a). Finally, the serving player is the one who covers the most distance per point, followed by the returning player, while partners of the serving and returning players are the ones who cover the least distance per point, both at the advanced and the beginner level (Ramón-Llín et al., 2020a, 2020c).

Finally, the adoption of different serving partner strategies also affects movements made by players. In this sense, a study by Ramón-Llin et al. (2021) analyses the distance covered by players when serving from the Australian position versus a traditional one. The results showed that the use of the Australian position forces the server to cover more distance and at a faster speed in his or her movement towards the net than when using a conventional position. On the other hand, when using a traditional position, in addition to covering less distance to the net, the server will be closer to the net and his or her side wall at the moment when the opposing player hits the return. These data may be related to the results that have shown a lower percentage of points won on the serve with the Australian position compared to the traditional one, since, at the moment of the third shot of the point, the server is further away from the net and his side wall, and the distance to the net, as explained above, is a variable that correlates significantly with the effectiveness of the shot (the further away, the more errors are made)

### Match Score Studies

Studies related to the score in padel have shown that, in men's padel, more than 70% of matches are played to 2 sets, while in women's padel this percentage drops to below 70% (Sánchez-Alcaraz et al., 2021b). Furthermore, in men's padel almost 50% of the sets are matched (score 6–4 or more), with a higher percentage of tie-breaks than in women's padel (Muñoz et al., 2022). It was also observed that in professional padel an average of almost 22 games are played per match, and almost 10 games per set, with significant differences between men's and women's padel (Muñoz et al., 2022).

However, the data related to the scoreboard varies from 2020 onwards, where the golden point rule was incorporated. A study carried out by Sánchez-Alcaraz et al. (2020c) has found that the use of the golden point results in slight reductions in set length (approx. 3 min), in addition to a higher number of breaks and more games per set and per match.In addition, more golden points are played in women's than in men's padel and, according to the number of sets, the number of golden points increases in the second set compared to the third set. On the other hand, the efficiency in gold points can determine the outcome of the match, as the winning pair of the match wins significantly more golden points than the losing pair (Muñoz et al., 2022). Finally, the round of the tournament also affects the match score, as it has been observed that as the rounds of play progress, the number of games per match increases, thus the equality also increases. Thus, both the physical and psychological demands in the final rounds seem to be greater than in previous rounds. Therefore, good physical preparation and the ability to cope with these sets and close matches could be an important key to success in the tournament.

## Limitations and Future Directions o Research

This study has certain limitations that should be taken into account when interpreting the results. Although the vast majority of the selected studies have a very large sample of strokes and actions, being representative for the analysis and pooling of results, others only analyze a small and specific number of players, which makes it difficult to compare the results between studies and does not allow to generalize the results. Furthermore, it has been observed that some performance analysis topics, such as the study of scoring, currently present very few studies, thus future research should evaluate these variables in greater depth.

## Conclusions

This is the first study that has carried out a systematic review of the analysis of performance in professional padel that brings together the research of the last five years. The results of these studies have made it possible to define the time structure of padel, its technical-tactical actions, the movements and distances covered by its players and the analysis of the scoreboard. These results have shown important differences in these four lines of research depending on the gender and the level of players, the side and the zone of play as well as the time of the match. In this way, the information gathered in this study will allow coaches, physical trainers and players to design training sessions based on the specific characteristics of padel, adapted to the demands of competition. Furthermore, it will contribute to the application of feedback and decision making based on statistics and match data.

## References

[ref1] Cartwright Hatton, S., Roberts, C., Chitsabesan, P., Fothergill, C., & Harrington, R. (2004). Systematic review of the efficacy of cognitive behaviour therapies for childhood and adolescent anxiety disorders. British Journal of Clinical Psychology, 43(4), 421–436. 15530212 10.1348/0144665042388928

[ref2] Courel-Ibáñez, J., Sánchez-Alcaraz, B.J., Garcia Benitez, S., & Echegaray, M. (2017a). Evolution of padel in Spain according to practitioners' gender and age. Cultura Ciencia y Deporte, 12(34), 39–46.

[ref3] Courel-Ibáñez, J., Sánchez-Alcaraz, J. B., & Cañas, J. (2015). Effectiveness at the net as a predictor of final match outcome in professional padel players. International Journal of Performance Analysis in Sport, 15(2), 632–640.

[ref4] Courel-Ibáñez, J., Sánchez-Alcaraz Martínez, B. J., & Cañas, J. (2017b). Game Performance and Length of Rally in Professional Padel Players. Journal of Human Kinetics, 55, 161–169. 10.1515/hukin-2016-0045 28210348 PMC5304268

[ref5] Courel-Ibáñez, J., Sánchez-Alcaraz, B. J., & Muñoz, D. (2019). Exploring Game Dynamics in Padel: Implications for Assessment and Training. Journal of Strength and Conditioning Research, 33(7), 1971–1977. 10.1519/JSC.0000000000002126 28723819

[ref6] Conde-Ripoll, R., Llanos, M.B., García, J.M., & Sánchez-Alcaraz, B.J. (2021). Wall stroke analysis in professional padel. Revista de Entrenamiento Deportivo, 35(2), 3–11.

[ref7] Díaz-García, J., González-Ponce, I., López-Gajardo, M. A., Manzano-Rodríguez, D., Lobo-Triviño, D., Rubio-Morales, A., & García-Calvo, T. (2023). How mentally fatiguing is play a semiprofessional padel competition? A study of gender differences. Padel Scientific Journal, 1(1), 7–22. 10.17398/2952-2218.1.7

[ref8] Díaz-García, J., González-Ponce, I., López-Gajardo, M. Á., Van Cutsem, J., Roelands, B., & García-Calvo, T. (2021). How Mentally Fatiguing Are Consecutive World Padel Tour Matches? International Journal of Environmental Research and Public Health, 18(17), 9059. 10.3390/ijerph18179059 34501648 PMC8431167

[ref9] Demeco, A., De Sire, A., Marotta, N., Spanò, R., Lippi, L., Palumbo, A., Iona, T., Gramigna, V., Palermi, S., Leigheb, M., Invernizzi, M., & Ammendolia, A. (2022). Match analysis, physical training, risk of injury and rehabilitation in padel: Overview of the literature. International Journal of Environmental Research and Public Health, 19(7), 4153. 35409836 10.3390/ijerph19074153 PMC8998509

[ref10] De Villarreal, E. S., Byrne, P. J., & Ramirez-Campillo, R. (2023). Change of Direction Ability as a Sensitive Marker of Adaptation to Different Training Configurations, and Different Populations: Results from Four Experiments. Journal of Human Kinetics, 85, 63–73. 10.2478/hukin-2022-0110 36643834 PMC9808807

[ref11] Escudero-Tena, A., Almonacid, B., Martínez, J., Martínez-Gallego, R., Sánchez-Alcaraz, B. J., & Muñoz, D. (2022a). Analysis of finishing actions in men's and women's professional padel. International Journal of Sports Science & Coaching, In Press. 10.1177/17479541221139970

[ref12] Escudero-Tena, A., Fernández-Cortes, J., García-Rubio, J., & Ibáñez, S. J. (2020). Use and Efficacy of the Lob to Achieve the Offensive Position in Women's Professional Padel. Analysis of the 2018 WPT Finals. International Journal of Environmental Research and Public Health, 17(11), 4061. 10.3390/ijerph17114061 32517261 PMC7312421

[ref13] Escudero-Tena, A., Muñoz, D., Sánchez-Alcaraz, B. J., García-Rubio, J., & Ibáñez, S. J. (2022b). Analysis of Errors and Winners in Men's and Women's Professional Padel. Applied Sciences, 12(16), 8125. 10.3390/app12168125

[ref14] Fernandez, A., & León-Prados, J. A. (2017). Technical and tactical assessment tool for padel. International Journal of Medicine and Physical Activity and Sport Sciences, 17(68), 693–714.

[ref15] García-Benítez, S., Courel-Ibáñez, J., Pérez-Bilbao, T., & Felipe, J. L. (2018). Game Responses During Young Padel Match Play: Age and Sex Comparisons. Journal of Strength and Conditioning Research, 32(4), 1144–1149. 10.1519/JSC.0000000000001951 29112057

[ref16] García-Giménez, A., Pradas de la Fuente, F., Castellar-Otín, C., & Carrasco-Páez, L. (2022). Performance Outcome Measures in Padel: A Scoping Review. International Journal of Environmental Research and Public Health, 19(7), 4395. 35410074 10.3390/ijerph19074395 PMC8998912

[ref17] Gea Garcí, G. M., Conesa Garre, C. M., Courel-Ibáñez, J., & Menayo Antúnez, R. (2021). Ball type and court surface: A study to determine the ball rebound kinematics on the padel wall. International Journal of Performance Analysis in Sport, 21(2), 226–241.

[ref18] Gómez Chacón, R., Pascua Barón, D., & Fernández Martínez, N. (2019). Evolution of the federative licenses (1994–2016). Paddle vs. tennis. Materiales para la historia del deporte, 16, 43–49.

[ref19] International Padel Federation. Rules of the game in padel the International Padel Federation (FIP), https://www.padelfip.com/es/ (2020, accessed 12 January 2023).

[ref20] Lupo, C., Condello, G., Courel-Ibáñez, J., Gallo, C., Conte, D., & Tessitore, A. (2018). Effect of gender and match outcome on professional paddle tennis competitions. RICYDE. International Journal of Sport Science, 14(51), 29–41. 10.5232/ricyde2018.05103.

[ref21] Mellado-Arbelo, O., Vidal, E.B., & Usón, M.V. (2019). Analysis of game actions in professional male padel. Cultura, Ciencia y Deporte, 14(42), 191–201.

[ref22] Muñoz, D., Toro-Román, V., Vergara, I., Romero, A., Fernández de Ossó Fuente, A. I., & Sánchez-Alcaraz, B. J. (2022). Analysis of the gold point and its relationship with performance in male and female professional padel players. Retos: Nuevas Tendencias En Educación Física, Deportes y Recreación, 45, 275–281. 10.47197/retos.v45i0.92388

[ref23] O'Donoghue, P. (2015). *An introduction to performance analysis of sport*. London and New York: Routledge Taylor & Francis Group.

[ref24] Pradas, F., Sánchez-Pay, A., Muñoz, D., & Sánchez-Alcaraz, B. J. (2021). Gender Differences in Physical Fitness Characteristics in Professional Padel Players. International Journal of Environmental Research and Public Health, 18(11), 5967. 10.3390/ijerph18115967 34199473 PMC8199605

[ref25] Ramón-Llín, J., Guzmán, J. F., Muñoz, D., Martínez-Gallego, R., Sánchez-Pay, A., & Sánchez-Alcaraz, B. J. (2022). Sequential analysis of final point strokes in padel using a decision tree. Revista Internacional de Medicina y Ciencias de la Actividad Física y del Deporte, 22(88), 933–947. 10.15366/rimcafd2022.88.013

[ref26] Ramón-Llín, J., Guzmán, J., Llana, S., Vuckovic, G., Muñoz, D., & Sánchez-Alcaraz Martínez, B. J. (2020a). Analysis of distance covered in padel based on level of play and number of points per match. Retos: Nuevas Tendencias En Educación Física, Deportes y Recreación, 39, 205–209. 10.47197/retos.v0i39.79322

[ref27] Ramón-Llin, J., Guzmán, J. F., Llana, S., Martínez-Gallego, R., James, N., & Vučković, G. (2019). The Effect of the Return of Serve on the Server Pair’s Movement Parameters and Rally Outcome in Padel Using Cluster Analysis. Frontiers in Psychology, 10, 1194. 10.3389/fpsyg.2019.01194 31191397 PMC6546820

[ref28] Ramón-Llin, J., Guzmán, J., Martínez-Gallego, R., Muñoz, D., Sánchez-Pay, A., & Sánchez-Alcaraz, B. J. (2020b). Stroke Analysis in Padel According to Match Outcome and Game Side on Court. International Journal of Environmental Research and Public Health, 17(21), 7838. 10.3390/ijerph17217838 33114684 PMC7662292

[ref29] Ramón-Llin, J., Guzmán, J., Martínez-Gallego, R., Muñoz, D., Sánchez-Pay, A., & Sánchez-Alcaraz, B.J. (2021a). Analysis of the situation on the court of the players in the serve and its relationship. Retos: Nuevas Tendencias En Educación Física, Deportes y Recreación, 41, 399–405.

[ref30] Ramón-Llin, J., Guzmán, J., Martínez-Gallego, R., Vučković, G., Muñoz, D., & Sánchez-Alcaraz, B. J. (2021b). Comparison of service tactic formation on players’ movements and point outcome between national and beginner level padel. Plos One, 16(10), e0250225. 10.1371/journal.pone.0250225 34705848 PMC8550361

[ref31] Ramón-Llin, J., Llana, S., Guzmán, J., Vuckovic, G., Muñoz, D., & Sánchez-Alcaraz, B. J. (2020c). Analysis of distance covered according to the player's strategic role and level. Acción Motriz, 25(1), 59–67.

[ref32] Sánchez-Alcaraz, B. J. (2013). History of paddle tennis. Materiales Para La Historia Del Deporte, 11, 57–60.

[ref33] Sánchez-Alcaraz, B.J., Cánovas, J., Sánchez-Pay, A., & Muñoz, D. (2023a). Research in paddle tennis. Systematic review. Padel Scientific Journal, 1(1), 71–105. 10.17398/2952-2218.1.7.

[ref34] Sánchez-Alcaraz, B. J., Cañas, J. & Courel-Ibáñez, J. (2015). Analysis of scientific research in paddle tennis. Agón, International Journal of Sport Sciences, 5(1), 44–54.

[ref35] Sánchez-Alcaraz, B. J., Courel-Ibáñez, J., Díaz, J., Grijota, F. J., & Muñoz, D. (2019). Effects of score difference and relevance of the point on temporal structure in first division padel matches. Journal of Sport and Health Research, 11(2), 151–160.

[ref36] Sánchez-Alcaraz, B. J., Courel-Ibáñez, J., Muñoz, D., Infantes-Córdoba, P., Sáenz de Zumarán, F., & Sánchez-Pay, A. (2020a). Analysis of attacking actions in professional men's paddle tennis. Apunts Educación Física y Deportes, 141, 29–34. 10.5672/apunts.2014-0983.es.(2020/4).142.04

[ref37] Sánchez-Alcaraz, B.J., Courel-Ibáñez, J., & Cañas, J. (2018). Temporal structure, court movements and game actions in padel: a systematic review. Retos: Nuevas Tendencias En Educación Física, Deporte y Recreación, 33, 308–312.

[ref38] Sánchez-Alcaraz, B. J., Jiménez, V., Muñoz, D., & Ramón-Llin, J. (2021a). External training load differences between male and female professional padel. Journal of Sport and Health Research, 13(3), 445–454.

[ref39] Sánchez-Alcaraz, B. J., Jiménez, V., Muñoz, D., & Ramón-Llin, J. (2022a). Efficiency and distribution of the final attacking strokes in professional padel. Revista Internacional de Medicina y Ciencias de la Actividad Física y del Deporte, 22(87), 635–648. 10.15366/rimcafd2022.87.013

[ref40] Sánchez-Alcaraz, B. J., Martínez-Gallego, R., Ramón-Llin Mas, J., Crespo, M., Muñoz, D., López Martínez, J. M., & Sánchez-Pay, A. (2022b). Professional padel tennis: Characteristics and effectiveness of the shots played to the fence. International Journal of Sports Science & Coaching, In Press. 10.1177/17479541221093765

[ref41] Sánchez-Alcaraz, B.J., Muñoz, D., Escudero-Tena, A., Martín-Miguel, I., & Martínez-García, J. (2023b). Hitting zone analysis in professional padel. Kronos, 21(2), 1–9.

[ref42] Sánchez-Alcaraz, B. J., Muñoz, D., Pradas, F., Ramón-Llin, J., Cañas, J., & Sánchez-Pay, A. (2020b). Analysis of Serve and Serve-Return Strategies in Elite Male and Female Padel. Applied Sciences, 10(19), 6693. 10.3390/app10196693

[ref43] Sánchez-Alcaraz, B. J., Muñoz, F. J., Ramón-Llin, J., Sánchez-Pay, A., & Muñoz, D. (2020c). Influence of golden point scoring in temporal structure and matches' outcome in professional padel. Kronos, 19(1), 1–8.

[ref44] Sánchez-Alcaraz, B.J., Muñoz, D., Sánchez-Pay, A., Martín-Miguel, I., Piedra, D., & Barriocanal, I. (2022c). Analysis of winning shots and errors in professional padel. Revista Iberoamericana de Ciencias de la Actividad Física y el Deporte, 11(3), 85–97.

[ref45] Sánchez-Alcaraz, B. J., Perez-Puche, D. T., Pradas, F., Ramón-Llín, J., Sánchez-Pay, A., & Muñoz, D. (2020d). Analysis of Performance Parameters of the Smash in Male and Female Professional Padel. International Journal of Environmental Research and Public Health, 17(19), 7027. 10.3390/ijerph17197027 32992940 PMC7579567

[ref46] Sánchez-Alcaraz, B. J., Siqueir-Coll, J., Toro-Román, V., Sánchez-Pay, A., & Muñoz, D. (2021b). Analysis of the parameters related to score in World Padel Tour 2019: differences by gender, round and tournament type. Retos: Nuevas Tendencias En Educación Física Deporte y Recreación, 39, 200–204.

[ref47] Sánchez-Alcaraz, B.J., & Courel-Ibáñez, J. (2022). The role of padel in improving physical fitness and health promotion: progress, limitations, and future perspectives-A narrative review. International Journal of Environmental Research and Public Health, 19(11), 6582. 35682167 10.3390/ijerph19116582 PMC9180804

[ref48] Sánchez-Muñoz, C., Muros, J. J., Cañas, J., Courel-Ibáñez, J., Sánchez-Alcaraz, B. J., & Zabala, M. (2020). Anthropometric and physical fitness profiles of world-class male padel players. International Journal of Environmental Research and Public Health, 17(2), 508. 31941164 10.3390/ijerph17020508 PMC7014060

[ref49] Villena-Serrano, M., Castro-López, R., & Zagalaz-Sánchez, M. (2020). Analysis of the subjective well-being of the paddle tennis player. Revista de Psicología del Deporte, 29(1), 29–37.

[ref50] Webster, J., & Watson, R. T. (2002). Analyzing the past to prepare for the future: Writing a literature review. MIS Quarterly, 26(2), xiii–xxiii.

[ref51] Wright, R., Brand, R., Dunn, W., & Spindler, K. (2007). How to write a systematic review. Clinical Orthopaedics and Related Research, 455, 23–29 17279036 10.1097/BLO.0b013e31802c9098

[ref52] Zırhlı, O., & Demirci, N. (2020). The Influence of functional training on biomotor skills in girl tennis players aged 10–12. Balt J Health Phys Activ, 12(4), 33-45. 10.29359/BJHPA.12.4.04

